# Evaluation of a virtual objective structured clinical examination in the metaverse (Second Life) to assess the clinical skills in emergency radiology of medical students in Spain: a cross-sectional study

**DOI:** 10.3352/jeehp.2025.22.12

**Published:** 2025-04-21

**Authors:** Alba Virtudes Perez-Baena, Teodoro Rudolphi-Solero, Rocio Lorenzo-Alvarez, Dolores Dominguez-Pinos, Miguel Jose Ruiz-Gomez, Francisco Sendra-Portero

**Affiliations:** 1 Department of Radiology, Hospital Comarcal de Antequera, Antequera, Spain; 2 Department of Radiology and Physical Medicine, Faculty of Medicine, University of Malaga, Malaga, Spain; 3 Department of Nuclear Medicine, Hospital Universitario Virgen de la Victoria, Malaga, Spain; 4 Department of Human Physiology, Faculty of Medicine, University of Malaga, Malaga, Spain; 5 Department of Emergency and Intensive Care, Hospital de la Axarquia, Velez-Malaga, Spain; 6 Department of Radiology, Hospital Universitario Virgen de la Victoria, Malaga, Spain; The Catholic University of Korea, Korea

**Keywords:** Medical students, Radiology, Computer simulation, Educational measurement, Spain, Cross-sectional studies

## Abstract

**Purpose:**

The objective structured clinical examination (OSCE) is an effective but resource-intensive tool for assessing clinical competence. This study hypothesized that implementing a virtual OSCE in the Second Life (SL) platform in the metaverse as a cost-effective alternative will effectively assess and enhance clinical skills in emergency radiology while being feasible and well-received. The aim was to evaluate a virtual radiology OSCE in SL as a formative assessment, focusing on feasibility, educational impact, and students’ perceptions.

**Methods:**

Two virtual 6-station OSCE rooms dedicated to emergency radiology were developed in SL. Sixth-year medical students completed the OSCE during a 1-hour session in 2022–2023, followed by feedback including a correction checklist, individual scores, and group comparisons. Students completed a questionnaire with Likert-scale questions, a 10-point rating, and open-ended comments. Quantitative data were analyzed using the Student t-test and the Mann-Whitney U test, and qualitative data through thematic analysis.

**Results:**

In total, 163 students participated, achieving mean scores of 5.1±1.4 and 4.9±1.3 (out of 10) in the 2 virtual OSCE rooms, respectively (P=0.287). One hundred seventeen students evaluated the OSCE, praising the teaching staff (9.3±1.0), project organization (8.8±1.2), OSCE environment (8.7±1.5), training usefulness (8.6±1.5), and formative self-assessment (8.5±1.4). Likert-scale questions and students’ open-ended comments highlighted the virtual environment’s attractiveness, case selection, self-evaluation usefulness, project excellence, and training impact. Technical difficulties were reported by 13 students (8%).

**Conclusion:**

This study demonstrated the feasibility of incorporating formative OSCEs in SL as a useful teaching tool for undergraduate radiology education, which was cost-effective and highly valued by students.

## Graphical abstract

[Fig f4-jeehp-22-12]

## Introduction

### Background/rationale

The objective structured clinical examination (OSCE) is a reliable, consistent, and reproducible evaluation method widely used in medicine and radiology [1-3]. It serves as both a summative and formative assessment, providing valuable feedback [4]. However, its implementation is costly, requiring dedicated spaces and resources, making virtual formats a promising alternative that warrants further exploration [4,5].

The metaverse is a virtual, computer-generated environment merging real and digital worlds into immersive 3-dimensional experiences. It includes 4 types [6]: augmented reality, which enhances real-life settings; lifelogging, which involves recording and sharing daily activities; mirror worlds, which replicate real-world environments; and virtual worlds, like Second Life (SL), which are fully immersive spaces for interaction, learning, and content creation. Advances in graphics and internet connectivity have made the metaverse globally accessible, enabling immersive 3-dimensional (3D) experiences, social interaction, and educational activities [7].

Launched in 2003 by Linden Lab, SL is one of the first metaverse platforms, providing a persistent virtual environment where users, as avatars, interact, create content, and engage in economic activities. Unlike traditional games, it lacks predefined objectives, letting users shape their experiences [8]. Studies highlight its role in medical education, particularly in promoting interactive and collaborative game-based learning experiences, with key advantages such as remote access, immersive experiences, ease of use, and free availability [7]. SL supports formative and summative OSCEs by enabling diverse clinical scenarios for decision-making training. Avatar-mediated OSCEs allow interaction with specific contexts, like geriatrics [9] or urology [10], but virtual radiology OSCEs in SL remain unexplored.

### Objectives

To address this gap in the literature, this study hypothesized that a virtual OSCE in SL can cost-effectively assess and improve clinical skills in emergency radiology, while being feasible and well-received. The aim was to evaluate a 3D virtual radiology OSCE in SL, focusing on feasibility, educational impact, and student perceptions by analyzing its training impact, skill evaluation, and user feedback.

## Methods

### Ethics statement

All data were processed anonymously in compliance with data protection laws. This study received approval from the Ethics Committee for Experimentation at the University of Málaga (CEUMA) under reference 141-2022-H on 18 January 2023.

### Study design

The authors conducted a cross-sectional study to assess the feasibility and educational impact of a virtual OSCE on emergency radiology in SL. Feedback was gathered from student performance in the OSCE, and a post-OSCE survey captured students’ perceptions and experiences. Results are presented in accordance with Strengthening the Reporting of Observational Studies in Epidemiology (STROBE) guidelines [11].

### Setting

This study was conducted from October 2022 to February 2023 with sixth-year medical students enrolled in a radiology rotation at the Faculty of Medicine of Málaga, Spain. The rotation lasted 10 working days and was conducted in 7 consecutive groups of 24 to 28 students. The virtual OSCE was held on “Medical Master Island,” a custom-designed space in SL (Supplements 1, 2). At the end of the OSCE, participants were invited to voluntarily complete a survey evaluating their perceptions of the experience.

### Intervention

**Fig. 1.**
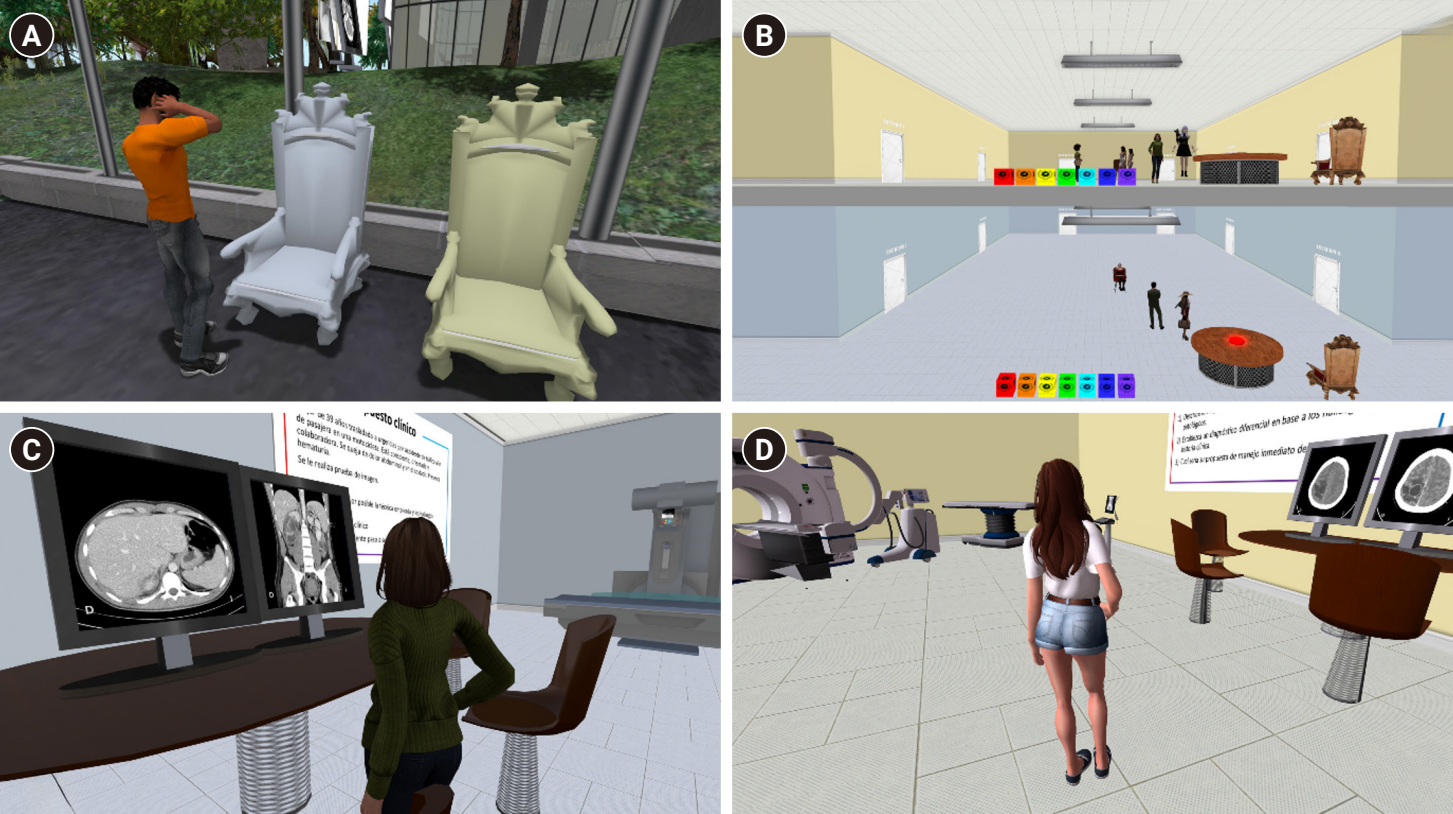
Various scenes from the virtual objective structured clinical examination (OSCE) experience in Second Life. (A) An avatar next to 2 chairs that teleport to the OSCE rooms. (B) View of the hallways of the Blue and Yellow OSCE rooms. (C) OSCE station in the Blue Room, showing a student viewing 2 monitors with abdominal computed tomography images. (D) OSCE station in the Yellow Room, showing a student reviewing neurology emergency case.

Two 6-station virtual OSCE rooms, the Blue Room and Yellow Room, were created; the rooms were located 565 meters apart vertically, allowing students to hear the instructor’s voice in both rooms. Each station simulated a medical or radiology consultation, featuring a wall panel with the clinical scenario and questions, along with a monitor displaying computed tomography (CT) images or X-rays (Fig. 1). Twelve emergency radiology cases were chosen to reflect common clinical scenarios, testing reasoning, imaging interpretation, and decision-making skills (Table 1). Faculty members validated the cases for curriculum alignment and relevance.

The OSCE accommodated groups of 12–14 students in 60–70-minute sessions, with 6 stations and a seventh rest station, enabling participation of up to 14 students simultaneously. Instructors provided audio and text instructions, such as “enter the station,” “2 minutes remaining,” and “exit the station,” as in conventional OSCEs. A faculty facilitator ensured adherence to the format and proper time management. After completing the OSCE, students received individualized feedback reports with performance scores and group comparisons, followed by an optional online survey to evaluate their perceptions of the virtual OSCE experience.

### Participants

In total, 172 sixth-year medical students (120 women and 52 men; mean age, 23.0±1.8 years) in the radiology rotation at the Faculty of Medicine of Málaga participated in the virtual OSCE sessions and were invited to complete the feedback survey. No exclusion criteria were applied.

### Variables

The feedback survey assessed efficiency, accessibility, clarity of instructions, tutor interaction, peer communication, format preferences, and overall satisfaction. Free-text responses were collected, allowing participants to add further comments.

### Data sources/measurement

The OSCE was scored using a checklist containing 8–10 items per station, with a total of 10 points per case. The checklist was developed by the authors (A.V.P.B. and T.R.S.) and validated by expert consensus with the involvement of the authors (M.J.R.G and F.S.P.; 2 faculty members with 20 and 40 years of academic experience, respectively) (Supplement 3). All evaluations were conducted by the first author (A.V.P.B.). The mean score across the 6 OSCE stations was calculated for each student. One week later, students received a self-assessment report detailing their item scores, station averages, and peer comparisons. The mandatory formative OSCE did not affect rotation grades, a point emphasized to help students identify strengths, weaknesses, and relative performance. The Pearson correlation between OSCE results and the final summative exam, involving 4 clinical radiology cases, was analyzed. The perception survey included: (1) a dichotomous question on prior familiarity with SL; (2) a 5-point Likert scale evaluating 13 project aspects (1=strongly disagree, 5=strongly agree); (3) cognitive load evaluation of 5 aspects using a 9-point scale [12]; (4) a 10-point rating for 9 project aspects; and (5) open-ended comments (Supplement 4). A 2-layer coding structure was used to analyze open-ended responses: first-layer codes (advantages, disadvantages, suggestions) and second-layer subcodes (Supplement 5). The OSCE results and survey responses are available from Dataset 1 and Dataset 2, respectively.

### Bias

The voluntary nature of the survey may have introduced response bias, as respondents might have held stronger opinions or greater interest in the virtual OSCE. To reduce this risk, students were invited to complete the survey anonymously and immediately after the OSCE. Standardization across sessions was ensured through a structured 6-station format administered uniformly for all groups. Another limitation is that the sample was restricted to a single university. Including students from other institutions would have strengthened the study by validating its conclusions and results.

### Study size

No formal study size calculation was performed, as all sixth-year medical students enrolled in the radiology rotation were included in the virtual OSCE sessions. The final sample represented over 94% of the target population, minimizing the risk of sampling bias and supporting the robustness of the findings.

### Statistical methods

Statistical analyses were conducted using IBM SPSS ver. 24.0 (IBM Corp.), with descriptive statistics compiled in Excel 2021 (Microsoft Corp.). Likert scale items were treated as ordinal variables, and OSCE scores as continuous variables, presented as mean±standard deviation (SD). The Student t-test was used to compare scores across groups and rooms, while the Mann-Whitney U test was used for non-parametric data (P<0.05). Free-text responses underwent thematic analysis. Two authors independently coded the responses to generate themes, which were collaboratively reviewed and refined.

## Results

### Participants

Of 172 students in the radiology rotation, 9 were excluded: 3 due to scheduling conflicts, 5 for technical failures (3 with insufficient computer capacity and 2 unable to render wall-panel images), and one for failing to submit OSCE responses. The final sample consisted of 163 students (113 women, 50 men), with a mean age of 23.5±1.8 years (median, 23 years; range, 22–38 years).

### Main results

**Fig. 2.**
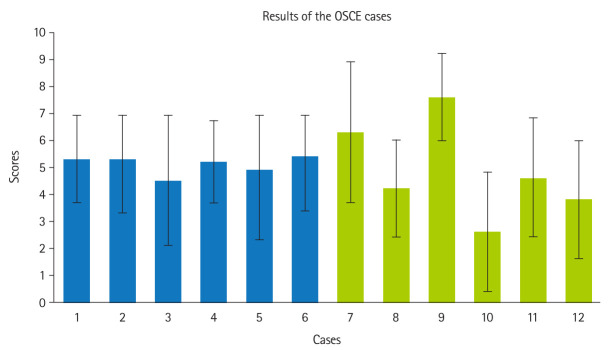
Bar chart representing the mean scores obtained in the 12 cases used in this experience. The colors correspond to the Blue and Yellow objective structured clinical examination (OSCE) rooms. Error bars indicate the standard deviation.

**Fig. 3.**
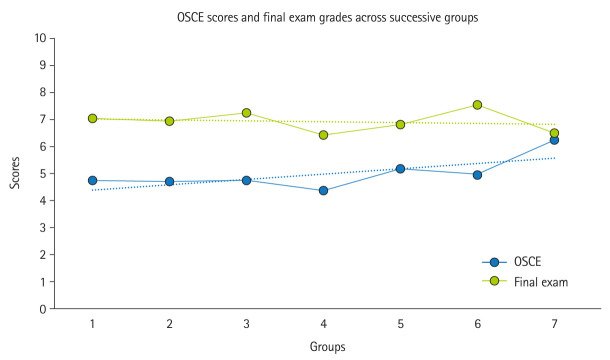
Objective structured clinical examination (OSCE) scores and final exam grades of the 7 groups that participated successively in the experience. The points represent the group means, and the dotted line indicates the linear regression.

The mean OSCE score was 5.0±1.4 out of 10 (median, 4.8; range, 2.3–9.3), with no significant differences observed between the Blue and Yellow rooms (5.1±1.4 vs. 4.9±1.3, P=0.287) or between men and women (5.0±1.3 vs. 5.0±1.5, P=0.728). Fig. 2 shows individual case scores, with the lowest mean score for acute diverticulitis on CT (2.6±2.2) and the highest for sigmoid volvulus on X-ray (7.6±1.6). The mean final exam grade was 6.9±1.3, significantly higher than the OSCE score (P<0.001), with a weak positive correlation between OSCE scores and final exam grades (Pearson coefficient=0.199, P=0.011). Fig. 3 displays OSCE scores and final exam grades across the 7 consecutive student groups.

A total of 117 students (71.8%) completed the evaluation questionnaire, with 42 providing open-ended comments. The survey revealed strong agreement regarding multiple aspects of the virtual OSCE (Table 2). Students rated the virtual environment highly attractive (4.5±0.8) and considered the selected cases suitable for training (4.2±1.0). Formative self-assessment received high ratings both for student interest (4.6±0.8) and utility in learning (4.5±0.9). Cognitive load analysis (Table 3) indicated low mental effort was required for movement and communication tasks in SL, whereas somewhat greater mental effort was reported for solving OSCE cases. Overall, the global experience was rated at 7.8±1.4 on a 10-point scale (Table 4), with teaching staff quality rated highest (9.3±1.0) and connectivity issues rated lowest (7.3±2.2). Thematic analysis of the 42 open-ended comments identified key themes categorized as advantages, disadvantages, and suggestions (Supplement 1, Table 5). Students praised the training value, innovation, and engaging experience, and expressed appreciation for the teaching staff’s guidance. Thirteen students (8%) reported technical difficulties, primarily due to outdated equipment or poor connectivity, and 8 students suggested providing clearer OSCE station instructions. Students also recommended adding detailed post-OSCE case explanations and enabling SL access from faculty computers to resolve technical limitations. Overall, student feedback highlighted strong interest and appreciation, reinforcing the virtual OSCE’s potential as an effective and innovative medical educational tool (Supplement 2).

## Discussion

### Key results

The virtual OSCE on emergency radiology was successfully implemented in SL, accommodating 12–14 students per 60–70-minute session with minimal resource use. Technical difficulties affected 8% of participants, but these issues were fully resolved. Student performance was moderate, with an average OSCE score of 5.0±1.4 out of 10. The formative self-assessment component was highly valued, as students appreciated receiving feedback to identify areas for improvement. Additionally, students expressed high satisfaction with the virtual format, highlighting its innovation, organization, teaching quality, and engaging nature.

### Interpretation

Our study explored the feasibility of using SL to evaluate students through a virtual OSCE. The virtual OSCE rooms received high ratings for attractiveness (mean score, 4.5±0.8) (Table 2). The absence of significant differences in scores between the Blue and Yellow rooms suggests that the case difficulty was comparable. Notably, OSCE scores showed improvement in successive student groups, with the final group achieving the highest scores (Fig. 3). This trend might reflect enhanced critical thinking developed during later rotations or potential information sharing among students. To minimize the risk of the latter, fostering a culture focused on formative assessment is essential, helping students understand that copying is unnecessary and counterproductive.

The high voluntary survey response rate (71.8%) indicates strong student interest in the project. Among the highly rated aspects, formative self-evaluation was particularly valued. Consistent with previous studies [13,14], students found formative OSCEs beneficial, especially appreciating detailed feedback from instructors. These findings underscore the value of formative self-assessments, and we recommend their inclusion in future OSCE projects to help students clearly identify weaknesses and enhance their learning.

The cognitive load analysis showed that communication and movement tasks in SL required minimal mental effort, reinforcing the platform’s suitability for formative OSCE implementation. Open-ended comments emphasized the project’s educational value, innovation, and enjoyable design. Students suggested incorporating additional OSCE case training to further enhance their learning experience. Although students expressed reservations about fully replacing in-person OSCEs—given that suitability may vary according to specific clinical skills assessed [5]—virtual OSCEs focusing on radiology reasoning can effectively complement traditional instruction. A formative virtual OSCE in SL is cost-effective in terms of infrastructure and logistics compared to equivalent in-person OSCEs, which require substantial investments in physical spaces and equipment. While the platform’s low monthly maintenance costs suggest logistical advantages, it should be noted that no formal economic analysis was conducted.

### Comparison with previous studies

In real-life settings, radiology-specific OSCEs have assessed medical students’ abilities to interpret images, diagnose conditions, and recommend management plans within a structured format, proving effective for radiology rotations [1,2]. Similar to this study, OSCEs often reveal clinical and radiological skill gaps overlooked by traditional clerkship evaluations [2]. We observed higher OSCE scores among groups completing the assessment later, possibly due to students sharing case information or answers, thus potentially compromising the assessment’s validity over time [2]. Rotation grades were poor predictors of individual OSCE scores, underscoring discrepancies between traditional summative evaluations and the formative OSCE [1]. Over the past decade, SL has proven to be a valuable, engaging, and enjoyable teaching tool in radiology education [7]. Its primary advantages—adequate image quality, remote access, a strong sense of presence, ease of use, and cost-free availability—underscore its value as a digital platform for conducting summative and formative OSCEs. Additionally, it facilitates the creation of diverse clinical scenarios for decision-making training. Although radiology OSCEs had not previously been developed in SL, prior studies highlight the potential for avatar-mediated OSCEs in medical education. Andrade et al. [9] demonstrated the feasibility of a 3D home-safety OSCE as an efficient alternative to traditional home visits for geriatric fellows. Similarly, Kava et al. [10] showed the effectiveness of a 3D virtual OSCE for assessing communication skills among urology residents, with participants praising its immersive and realistic nature. Both studies, however, were limited by small sample sizes (8–12 learners). By contrast, our study involved a larger cohort, enabling more robust analysis and greater generalizability.

### Limitations

This study has several limitations. First, technical challenges such as outdated computers or poor internet connections occasionally interfered with interaction in SL, although these issues typically affected fewer than 10% of participants [7]. Second, adapting to the SL platform required additional time and effort from students and instructors, and creating virtual content involved substantial initial effort, although this burden could be mitigated by repurposing existing materials. Third, the study was conducted at a single Faculty of Medicine, limiting the generalizability of findings despite their promising nature. Moreover, progressively increasing OSCE scores across sequential groups may indicate a learning effect or unintended case sharing; future studies with randomized group allocation would help minimize this potential bias. Lastly, the virtual OSCE exclusively focused on emergency radiology, limiting the range of clinical competencies assessed.

### Generalizability

The virtual OSCE stations developed for this project proved feasible in SL as a formative educational tool. Expanding their use to broader radiology courses or students at other universities is a promising direction, currently under development.

### Suggestions

Several improvements could optimize this educational approach. Increasing the number of OSCE stations would allow more students to participate simultaneously. Verifying reproducibility of checklist-based scoring, as noted by Staziaki et al. [2], could enhance assessment objectivity. Integrating artificial intelligence could automate scoring, reduce evaluator workload, and accelerate feedback provision. Student engagement might be strengthened through external incentives, such as inter-university competitions or recognition for high performance. Future studies should adopt randomized group designs to compare the SL platform with 2-dimensional alternatives (e.g., Moodle) to address potential group-related bias. To improve accessibility, institutional computers could have pre-installed SL viewers. Lastly, expanding the program to include family medicine or radiology residents would enable comparisons across experience levels and support longitudinal tracking of skill development.

### Conclusion

This study introduced a novel online formative OSCE by developing radiological stations in SL. The method proved useful and cost-effective, demonstrating feasibility within the virtual environment and effectiveness in formative teaching. Students praised its educational value, appreciating its innovative and well-organized design. They found the approach engaging and recommended incorporating additional OSCE case training. Further research into its application at undergraduate and postgraduate levels, along with expanding the sample size through multicenter studies, would yield valuable insights.

## Figures and Tables

**Figure f4-jeehp-22-12:**
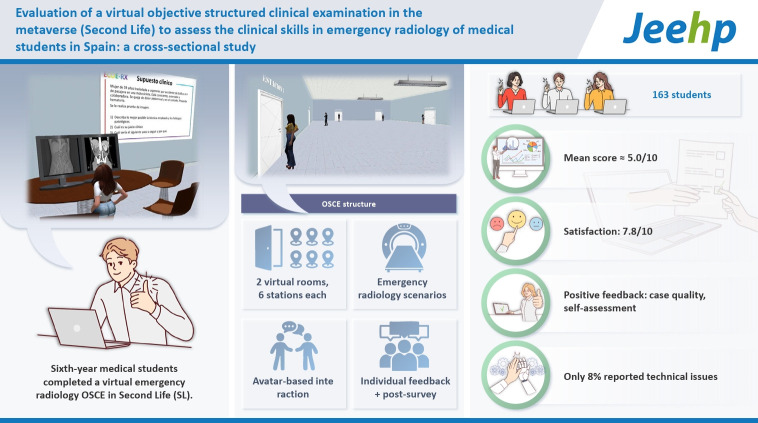


**Table 1.** Overview of emergency radiology cases from OSCE stations by imaging modality and clinical presentation

Room station (S)	Case no.	Image modality	Clinical presentation
Blue S1	1	Brain CT without IV contrast: 18 axial slices on one screen.	52-year-old man. Spontaneous intraparenchymal hemorrhage with a subarachnoid component.
Blue S2	2	Abdominal CT with IV contrast: 18 axial slices on one screen and 18 coronal slices on another screen.	39-year-old woman. Multiple renal lacerations with hemoperitoneum.
Blue S3	3	Chest X-ray: posteroanterior.	65-year-old woman. Complete opacification of the left hemithorax: atelectasis of left lung due to a bronchial carcinoma.
Blue S4	4	Brain CT without IV contrast: 18 axial slices on one screen.	79-year-old man. Acute ischemic lesion in the left cerebral hemisphere: MCA stroke with subfalcine herniation.
Blue S5	5	Abdominal CT with IV contrast: 18 axial slices on one screen.	55-year-old woman. Gallbladder distention, wall thickening, mucosal hyperenhancement, and pericholecystic fat stranding: acute cholecystitis.
Blue S6	6	Brain CT without IV contrast: 18 axial slices on one screen and 18 axial slices in bone window on another screen.	40-year-old man. Parietal acute epidural hematoma with associated bone fracture.
Yellow S1	7	Abdominal CT with IV contrast: 18 axial slices on one screen.	55-year-old man. Ruptured abdominal aortic aneurysm with hemoperitoneum.
Yellow S2	8	Brain CT without IV contrast: 18 axial slices on one screen and 18 axial slices in bone window on another screen.	58-year-old man. Frontal intraparenchymal hemorrhage, subdural hemorrhage, and occipital skull fracture.
Yellow S3	9	Antero-posterior abdominal radiography	73-year-old man. Large bowel dilatation with coffee bean sign: acute sigmoid volvulus with pneumoperitoneum.
Yellow S4	10	Abdominal CT with IV contrast: 18 axial slices on one screen.	60-year-old man. Colonic wall thickening, pericolic fat stranding in an area of sigmoid diverticulosis: acute diverticulitis.
Yellow S5	11	Brain CT with and without IV contrast: 18 axial slices on one screen and 18 axial slices on another screen.	55-year-old woman. Single cortical lesion, round, well-demarcated with enhancement and perilesional vasogenic edema: metastatic lesion.
Yellow S6	12	Abdominal CT with IV contrast: 18 axial slices on one screen and 18 coronal slices on another screen.	49-year-old man. Large soft-tissue mass, with internal heterogeneity: retroperitoneal sarcoma.

OSCE, objective structured clinical examination; CT, computed tomography; IV, intravenous; MCA, middle cerebral artery.

**Table 2.** Participant agreement levels on key aspects of the OSCE experience in Second Life

Experience in the project	Blue Room	Yellow Room	P-value	All
Your computer runs properly in Second Life without problems.	4.4±1.0	3.7±1.2	0.000	4.0±1.2
Your internet connection allows you to work in Second Life.	4.6±0.8	4.0±1.1	0.002	4.3±1.0
The environment of the OSCE room was attractive.	4.5±0.9	4.5±0.7	0.941	4.5±0.8
The design of the OSCE was appropriate for you.	4.4±1.0	4.2±1.0	0.181	4.3±1.0
The prior information of OSCE was appropriate for you.	4.4±1.1	4.4±1.0	0.981	4.4±1.0
The selection of OSCE cases was appropriate for your training.	4.4±0.9	4.0±1.0	0.016	4.2±1.0
The formative self-assessment was interesting for you.	4.6±0.9	4.6±0.6	0.914	4.6±0.8
The formative self-assessment was useful to learning.	4.6±0.9	4.4±0.9	0.197	4.5±0.9
You agreed with the mark obtained in your self-assessment.	4.4±1.0	4.4±0.7	0.885	4.4±0.8
You had fun in this experience.	4.0±1.1	4.0±0.9	0.932	4.0±1.0
Learning radiology in Second Life seems interesting to you.	4.2±1.0	4.1±1.0	0.479	4.2±1.0
You would participate in another virtual OSCE in Second Life.	3.7±1.2	3.5±1.1	0.470	3.6±1.2
You would participate in another Second Life experience when you are a resident.	3.8±1.1	3.5±1.1	0.205	3.7±1.1

Values are presented as mean±standard deviation on a 5-point Likert scale: 1=totally disagree; 2=disagree; 3=neither agree nor disagree; 4=agree; 5=totally agree. P-value represents the probability of error from the Mann-Whitney U test comparing Blue Room and Yellow Room results. OSCE, objective structured clinical examination.

**Table 3.** Evaluation of cognitive load across different tasks in the OSCE in Second Life

Cognitive load	Blue Room	Yellow Room	P-value	All
Move or scroll in Second Life	3.6±2.0	3.4±2.2	0.651	3.5±2.1
Communicate via written chat	2.6±2.0	2.4±1.8	0.547	2.5±1.9
Communicate via voice	3.1±2.0	3.0±2.2	0.819	3.0±2.1
Editing and costume tasks of your avatar	5.0±2.4	4.7±2.3	0.524	4.9±2.4
Resolve the OSCE cases in virtual rooms	6.4±1.7	6.2±2.0	0.545	6.3±1.9

Values are presented as mean±standard deviation on a 9-point Likert scale, where: 1=very, very low mental effort; 2=very low mental effort; 3=low mental effort; 4=somewhat low mental effort; 5=neither much nor little mental effort; 6=somewhat high mental effort; 7=high mental effort; 8=very high mental effort; and 9=very, very, very high mental effort. P-value represents the probability of error from the Mann-Whitney U test comparing Blue Room and Yellow Room results. OSCE, objective structured clinical examination.

**Table 4.** Student global ratings of key project aspects (0–10 scale)

Qualification of the project	Blue Room	Yellow Room	P-value	All
Global experience	7.8±1.5	7.8±1.3	0.847	7.8±1.4
Organization of the project	8.8±1.1	8.8±1.2	0.996	8.8±1.2
Environment of the OSCE stations	8.8±1.4	8.6±1.5	0.545	8.7±1.5
OSCE cases	8.2±1.3	7.7±1.6	0.080	8.0±1.5
The formative self-assessment	8.6±1.3	8.5±1.4	0.610	8.5±1.4
The professors	9.3±1.0	9.2±1.0	0.598	9.3±1.0
Utility for your training	8.7±1.5	8.5±1.5	0.455	8.6±1.5
Interaction with peers	8.1±2.0	7.9±2.1	0.656	8.0±2.1
Connectivity to Second Life	7.7±2.0	6.9±2.2	0.036	7.3±2.2

Values are presented as mean±standard deviation. P-value represents the probability of error from the Student t-test for unpaired data comparing Blue Room and Yellow Room results. OSCE, objective structured clinical examination.

**Table 5.** Key subcodes for first-level codes (advantages, disadvantages, and suggestions) in open-ended student feedback

Subcode	Explanation	No.
Advantages		
Appreciation	General positive feedback using terms/phrases like “I liked it,” “interesting,” “attractive,” “gratifying,” “positive,” “very cool,” “fantastic,” and “great.”	21
Didactic	Comments highlighting the experience as beneficial for learning, describing it as “useful,” “formative,” “educative,” and having a “good objective.”	14
Acknowledgments	Expressions of gratitude toward professors for their effort, design, and organization of the OSCE.	12
Innovation	Noting the experience as “new,” “original,” “unusual,” “surprising,” “creative,” and a “suitable alternative” to traditional learning methods.	8
Fun	Describing the activity as “fun,” “entertaining,” and “pleasant.”	6
Cases	Positive feedback on the interest level, balanced difficulty, and didactic value of the OSCE cases for active learning.	5
Organization	Reflections on the adequacy of guidelines, and the clarity and sufficiency of the provided information and organization.	4
Formative	Comments on the usefulness, interest, and formative nature of the self-assessment process in enhancing learning.	2
Stress management	Recognizing the OSCE as a helpful tool for managing stress and time while solving cases.	2
Platform 3D	Preference for this 3D platform over traditional 2D platforms, citing better communication between teachers and students.	1
Disadvantages		
Technical problems	Issues related to obsolete equipment, internet connectivity, or improper functioning of Second Life.	13
Information	Complaints about insufficient information regarding OSCE stations and their operation.	8
Second Life	Descriptions of Second Life as complicated, stressful, or impractical to use.	6
Time	Concerns about the activity being time-consuming, particularly in the final year of studies due to heavy workloads.	3
Platforms 2D	Preference for 2D online platforms, such as the Faculty of Medicine’s virtual campus, over Second Life.	2
Cases	Criticisms about the difficulty of the OSCE cases or the complexity of the questions.	2
Suggestions		
Cases	Suggestions to provide additional practice cases and more detailed case explanations to enhance preparation for the final summative OSCE at the end of the curriculum.	3
Other platforms	Recommendations to use alternative platforms when Second Life fails or faces connectivity issues.	1
Voluntary	Suggestions to make these activities voluntary rather than mandatory	1
Computers with Second Life	Proposals to enable Second Life on computers in the Faculty of Medicine library for easier access.	1

OSCE, objective structured clinical examination; 3D, 3-dimensional; 2D, 2-dimensional.

## Footnotes

**Data availability**
Data files are available from Harvard Dataverse: https://doi.org/10.7910/DVN/6FZGH9 Dataset 1. Raw data of the OSCE results.

Dataset 2. Raw data of the questionnaire.

## Supplementary materials

Supplementary files are available from Harvard Dataverse: https://doi.org/10.7910/DVN/6FZGH9

Supplement 1. Video showing student interaction with virtual OSCE cases in Medical Master Island.

Supplement 2. Detailed description of the Virtual OSCE program.

Supplement 3. Questionnaires used for evaluation of a virtual objective structured clinical examination.

Supplement 4. List of open-ended comments included in the survey.

Supplement 5. Code domains of open-ended comments with examples and illustrative quotes.
