# Supplementary material for: Evaluation of a virtual objective structured clinical examination in the metaverse (Second Life) to assess the clinical skills in emergency radiology of medical students in Spain: a cross-sectional study
Source: J Educ Eval Health Prof. 2025 Apr 21;22:12. doi: 10.3352/jeehp.2025.22.12 (PMC12202975; doi:10.3352/jeehp.2025.22.12)
Supplement: Supplementary file 4 — Supplement 2. Detailed description of the Virtual OSCE program. [file jeehp-22-12-suppl2.docx]

**Supplement 2: Detailed Description of the Virtual OSCE Program**

**Contents**

[1. Platform Description 1](#_Toc195281258)

[2. Design of the Virtual OSCE 1](#_Toc195281259)

[3. Technical Setup 2](#_Toc195281260)

[4. Organization of OSCE Sessions 2](#_Toc195281261)

[5. Clinical Cases 2](#_Toc195281262)

[6. Evaluation and Feedback 3](#_Toc195281263)

[7. Student Interaction & Communication 3](#_Toc195281264)

[8. Challenges & Considerations 3](#_Toc195281265)

[9. Suggestions for Reproducibility 3](#_Toc195281266)

[10. Screenshots of the OSCE stations and environment 4](#_Toc195281267)

[11. Example of an Assessment report sent to a student (translated into English) for formative feedback 6](#_Toc195281268)

# 1. Platform Description

- **Name:** Second Life (SL), developed by Linden Lab. [www.secondlife.com](http://www.secondlife.com)
- **Access method:** Desktop client (Second Life Viewer).
- **Environment:** Custom-designed virtual island called "Medical Master Island."

<https://maps.secondlife.com/secondlife/Medical%20Master%20Island/121/87/23>

- **Rationale for use:** SL is a free and immersive 3D platform that supports real-time avatar interaction, suitable for simulating clinical scenarios for formative assessments.

# 2. Design of the Virtual OSCE

- **Structure:** Two independent OSCE rooms named the "Blue Room" and the "Yellow Room," each with six clinical stations.
- **Separation:** Rooms were vertically separated by 565 meters, allowing instructors' voice to reach both simultaneously.
- **Each Station Included:**
  - A wall panel displaying the clinical scenario and specific questions.
  - A monitor showing diagnostic images (CT scans or X-rays).
  - Seating for the student's avatar.
- **Rest station:** A seventh, non-evaluative rest station was included to accommodate up to 14 participants.

# 3. Technical Setup

- **Minimum Requirements:**
  - Adequate RAM and GPU to support 3D rendering.
  - Stable high-speed internet connection.
  - Pre-installed Second Life Viewer software.
- **Instructor Tools:**
  - Voice communication (local range).
  - Text chat for announcements and instructions.
  - Control over student flow and timing within the environment.

# 4. Organization of OSCE Sessions

- **Participants per session:** 12 to 14 sixth-year medical students.
- **Session duration:** Approximately 60–70 minutes.
- **Schedule:** Seven student groups participated between October 2022 and February 2023.
- **Flow:**
  - Students teleported to the OSCE area.
  - Instructors guided progression with timed voice and text prompts.
  - Each station: ~6 minutes.
  - Movement between stations mirrored traditional in-person OSCEs.

# 5. Clinical Cases

- **Total cases:** 12 (6 per room).
- **Focus:** Emergency radiology, including trauma, stroke, acute abdomen, and other urgent pathologies.
- **Case Design:**
  - Selected for curricular relevance.
  - Validated by radiology faculty for level-appropriateness and clinical balance.
- **Modalities used:** Brain CT, Abdominal CT, Chest X-ray, Abdominal radiography.

# 6. Evaluation and Feedback

- **Scoring system:**
  - Checklist with 8–10 items per case (maximum score = 10).
  - Developed by academic staff and validated by experienced faculty.
- **Assessment:** Conducted by the same evaluator (first author) across all sessions.
- **Feedback:**
  - Personalized reports provided one week after the OSCE.
  - Included individual station scores and group performance comparisons.

# 7. Student Interaction & Communication

- **Instructor communication:** Primarily via voice; reinforced through written chat.
- **Student actions:**
  - Avatars navigated to each station independently.
  - Interaction was limited to viewing and analyzing content per station.
- **Peer interaction:** Minimal, due to time-structured and individually focused assessment.

# 8. Challenges & Considerations

- **Technical difficulties:**
  - Affected ~8% of students (mostly due to outdated hardware or weak internet).
  - Mitigated through prior instructions and availability of backup access options.
- **Learning curve:**
  - An optional tutorial was offered to familiarize students with the platform.
  - Instructors monitored adaptation and provided real-time assistance.

# 9. Suggestions for Reproducibility

- Use modular, pre-built OSCE environments that can be cloned across institutions.
- Offer preparatory sessions to ensure familiarity with the platform.
- Assign one technical support member per session.
- Ensure clinical cases and checklists are peer-reviewed and curriculum-aligned.

# 10. Screenshots of the OSCE stations and environment

**
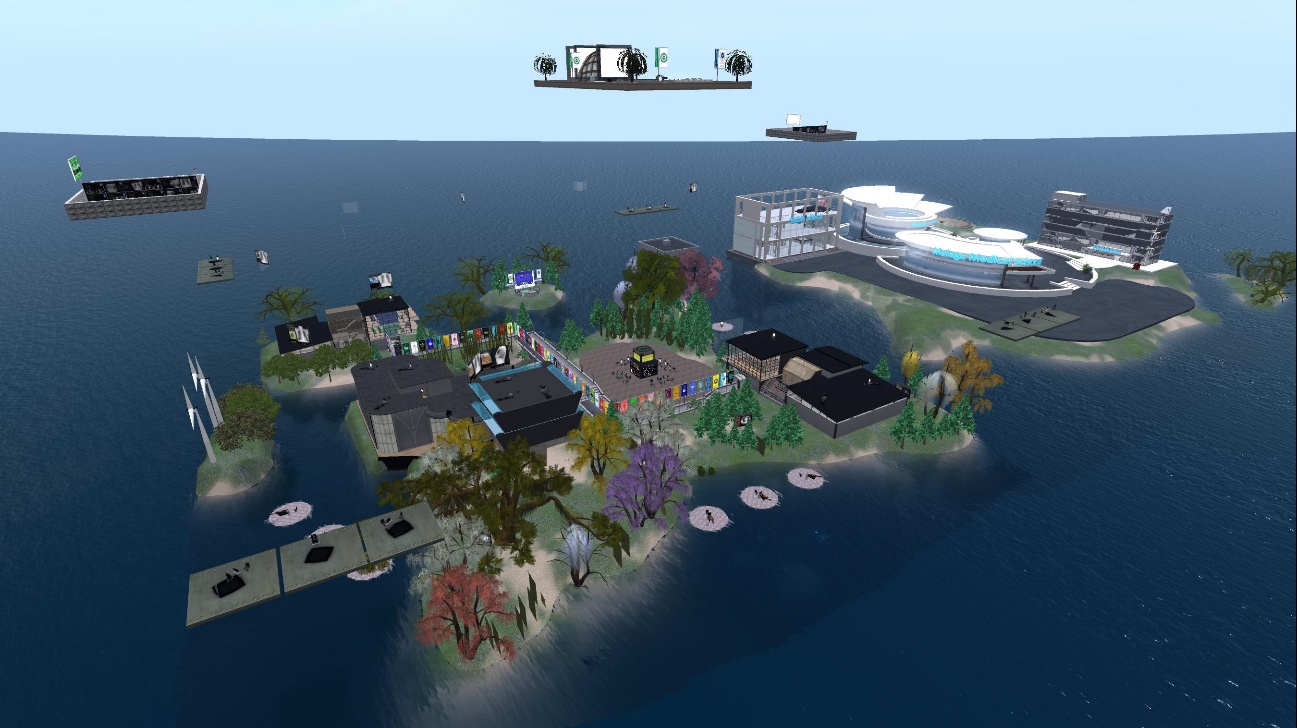
**

**Fig. S-1. Aerial view of the Medical Master Island.**


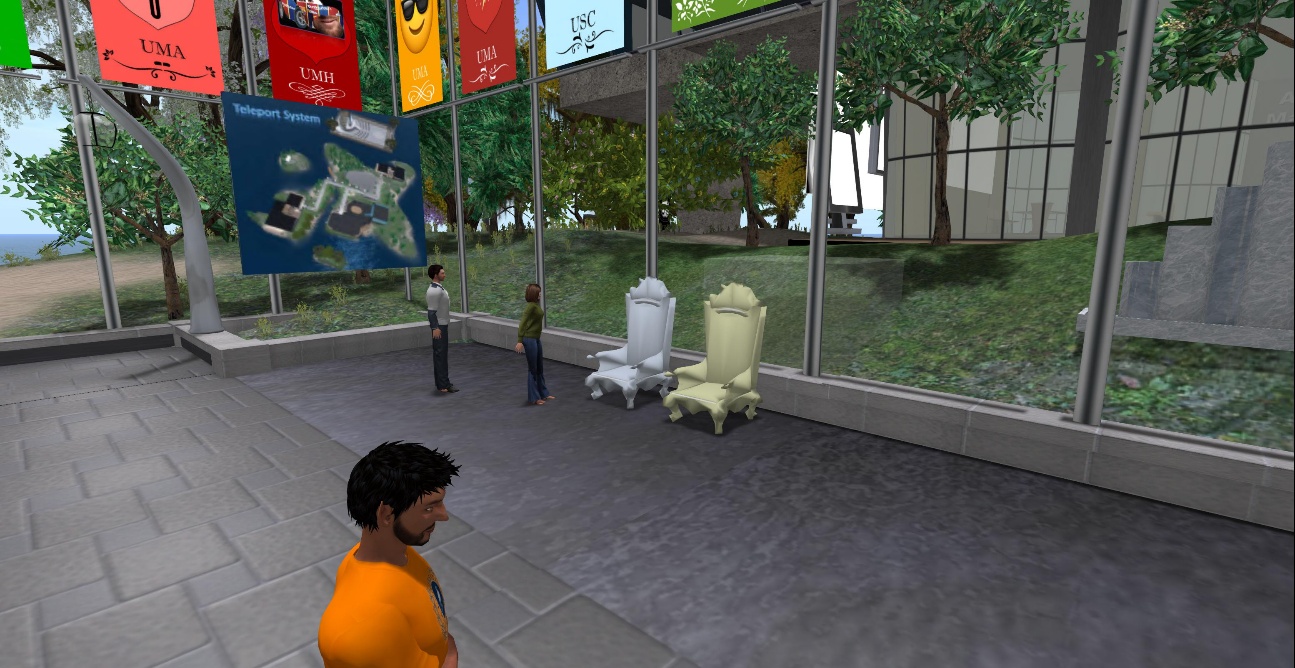


**Fig. S-2. Students at the island’s access point, in front of the chairs that teleport them to the Blue and Yellow OSCE rooms.**

**
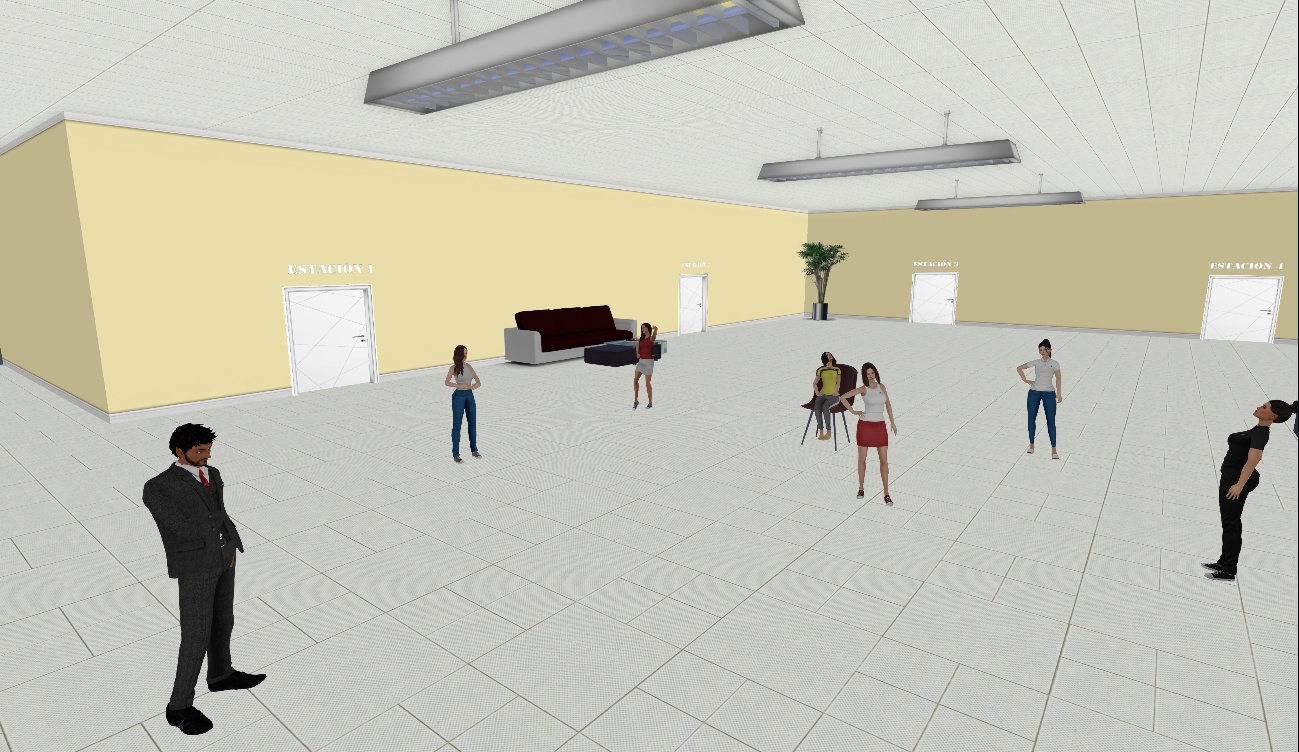
**

**Fig. S-3. Students in the Yellow OSCE room, just before starting the OSCE exam.**

**
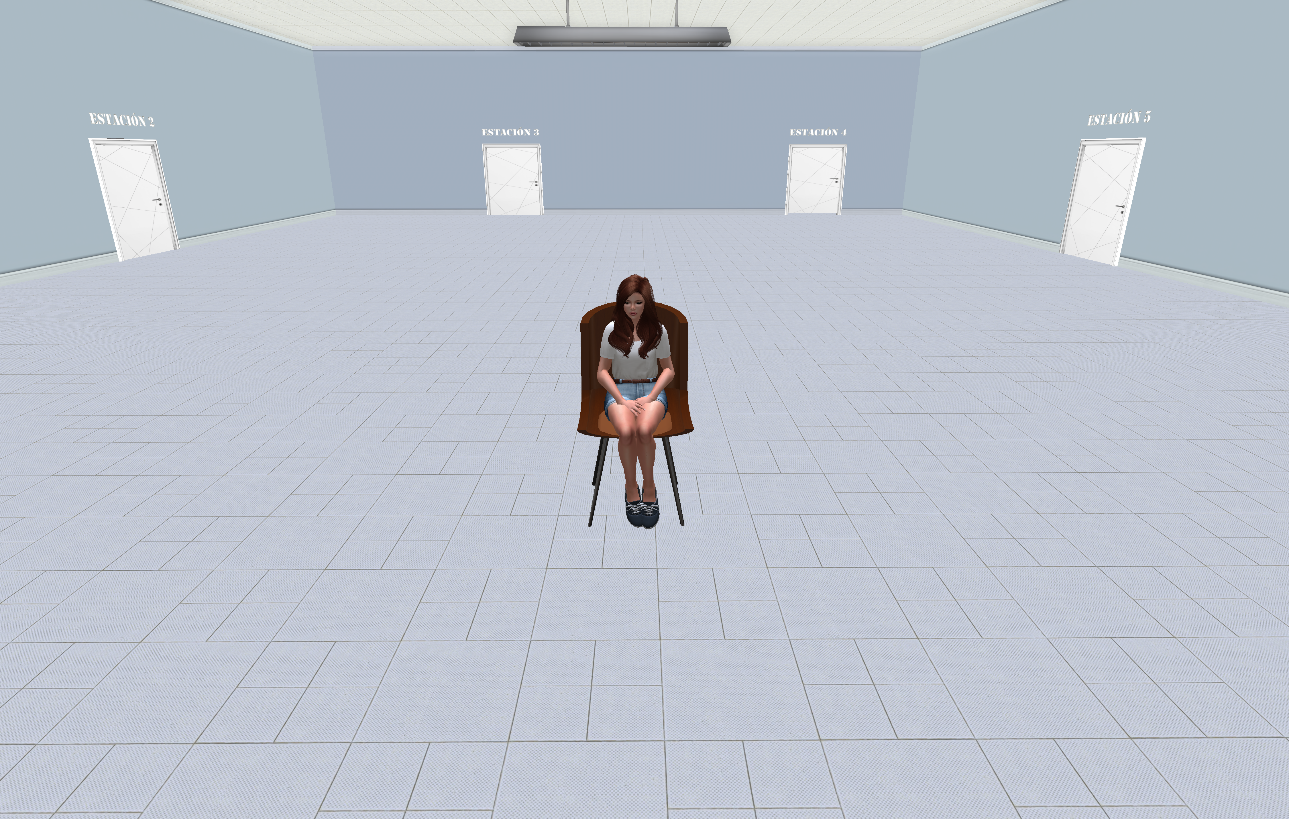
**

**Fig. S-4. Student at the rest station in the Blue OSCE room, while the other students are engaged in the remaining six stations.**

**
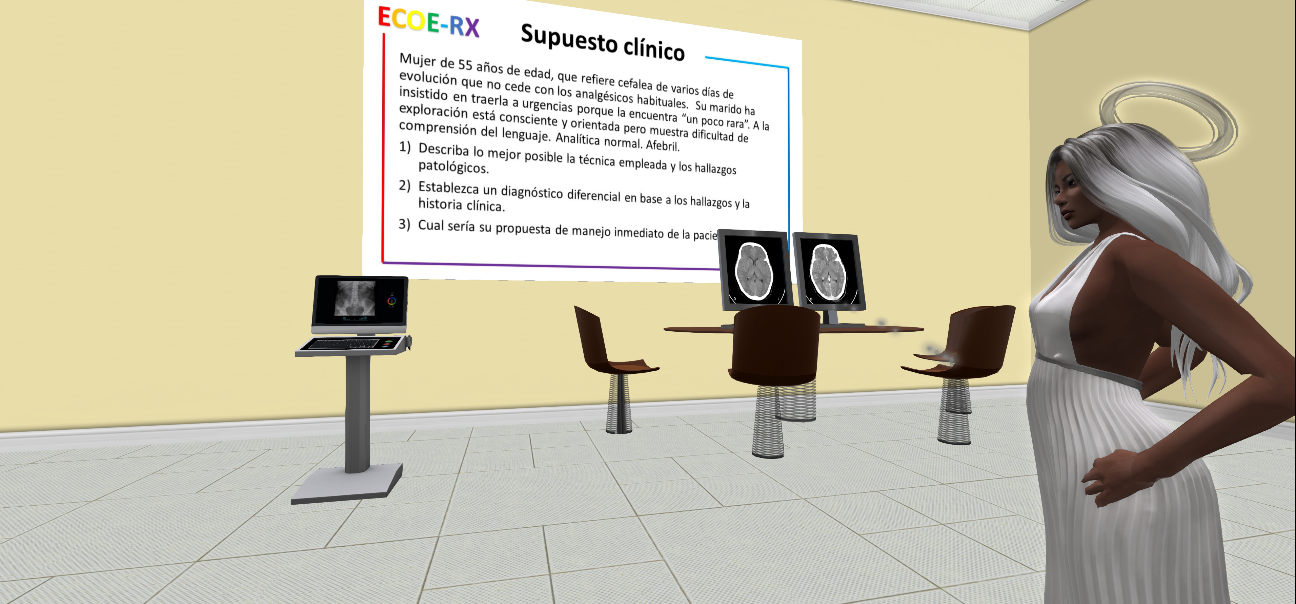
**

**Fig. S-5. Student at an OSCE station in the Yellow room, facing a clinical case with two monitors displaying CT images of the head.** **The poster on the wall displays the clinical scenario with the case description and the questions to be answered.**


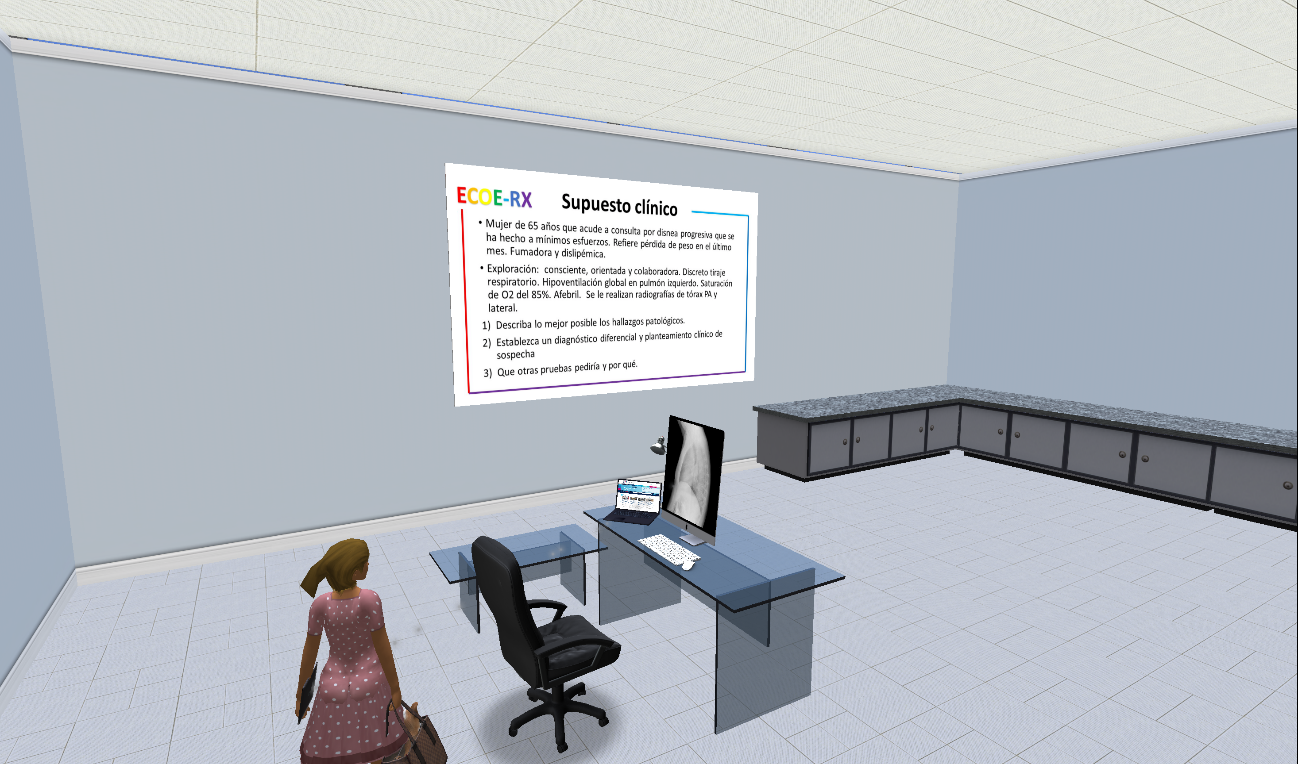


**Fig. S-6. Student at a station in the Blue OSCE room, working on a case involving a chest X-ray.**

# 11. Example of an Assessment report sent to a student (translated into English) for formative feedback


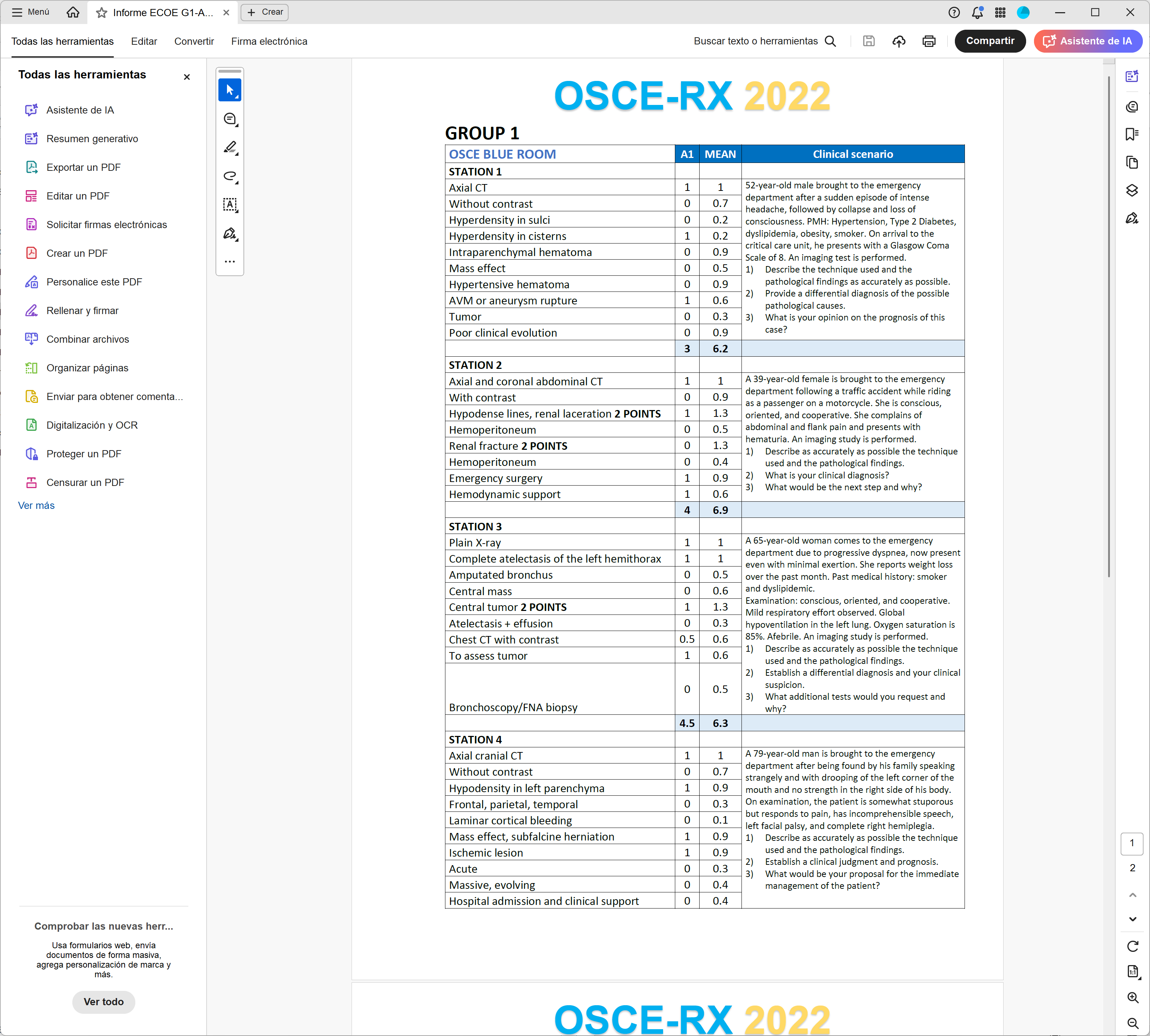


**Page 1 of 2**


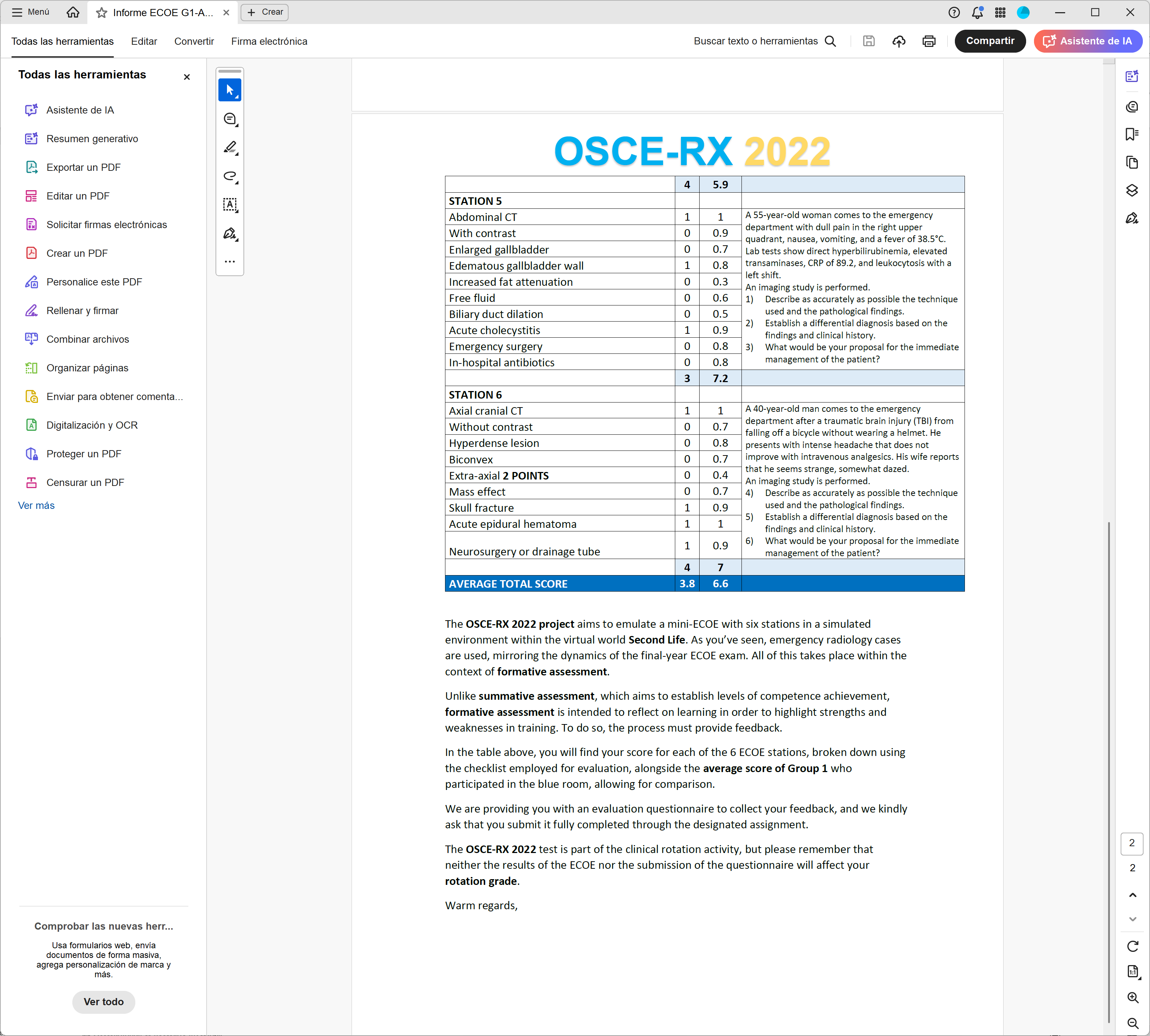


**Page 2 of 2**
